# Increased C4 and decreased C3 levels are associated with a poor prognosis in patients with immunoglobulin A nephropathy: a retrospective study

**DOI:** 10.1186/s12882-017-0658-7

**Published:** 2017-07-11

**Authors:** Min Pan, Ji Zhang, Zhanyuan Li, Lingwei Jin, Yu Zheng, Zhihong Zhou, Su Zhen, Guoyuan Lu

**Affiliations:** 1 Department of Nephrology, The First Affiliated Hospital of Soochow University, 88 Shizi St., Suzhou, 215006 Jiangsu People’s Republic of China; 2 Department of Nephrology, The Second Affiliated Hospital & Yuying Children’s Hospital of Wenzhou Medical University, Wenzhou, Zhejiang People’s Republic of China; 3 Department of Nephrology, The First Affiliated Hospital of Wenzhou Medical University, Wenzhou, Zhejiang People’s Republic of China

**Keywords:** IgA nephropathy, Complement, Chronic kidney disease, Renal failure

## Abstract

**Background:**

An association between serum complement levels and poor renal prognosis in patients with immunoglobulin A nephropathy (IgAN) remains controversial.

**Methods:**

We conducted a retrospective study examining the relationship between serum complement levels and prognosis in patients with IgAN. Between 2009 and 2013, patients with biopsy-confirmed IgAN were identified from the Second Affiliated Hospital of Wenzhou Medical College, China, and various parameters were documented during follow-up until 2015. The definition of the primary endpoint was a decrease of estimated glomerular filtration rate (eGFR) more than 30% from their baseline levels.

**Results:**

A total of 403 patients (55.3% female, average 33.7 months of follow-up) were identified and enrolled, with the primary endpoint occurring in 39 (9.8%) patients. Among the patients selected, 202 (50.1%) received corticosteroid treatment alone or in combination with another immunosuppressant (GS group), while others did not receive immunosuppressive treatment (non-GS group). The incidence of the primary endpoint was slightly lower in the GS group compared to the non-GS group (7.0% versus 12.6%, *p* = 0.06). Multivariate Cox proportional-hazard regression analyses, adjusting for age, systolic and diastolic blood pressure, 24-h urine protein, and immunosuppressive therapy, showed that serum complement 4 (C4) levels (hazard ratio [HR] 2.4, 95% confidence interval [CI] 1.6-3.8, *p* < 0.001) and serum complement 3 (C3) levels (HR 0.6, 95% CI 0.2-0.6, *p* < 0.001) were significantly associated with a poor prognosis among patients with IgAN.

**Conclusions:**

We demonstrated that an increase in serum C4, as well as a decrease in C3, was an important outcome determinant for patients with IgAN. Testing serum C3 and C4 levels might assist in predicting renal outcomes among these patients.

## Background

Immunoglobulin A nephropathy (IgAN) is the most common type of primary glomerulonephritis worldwide, and is characterized by the deposition of IgA in the glomerular mesangium [1, 2]. The clinical course of IgAN is highly variable, ranging from asymptomatic disease with abnormal urinalysis findings, to rapidly progressive renal failure. Approximately one-third of patients with IgAN eventually develop end-stage renal disease (ESRD) within 20–30 years after their initial diagnosis [1].

Over the last two decades, risk factors for the progression of IgAN have been identified, including the presence of proteinuria, high blood pressure, and low estimated glomerular filtration rate (eGFR) at the time of renal biopsy. Findings from pathological examination are also found to be associated with renal outcomes; for example, a new grading system termed Oxford Classification of IgAN, as well as the MEST scoring system, attempt to score changes in different anatomical compartments including mesangium hypercellularity (M), endocapillary hypercellularity (E), segmental glomerulosclerosis (S), and tubular atrophy/interstitial fibrosis (T). Results obtained using this classification system has been validated in a variety of studies for the prediction of renal outcomes [3, 4]. However, the aforementioned parameters may fail to capture the degree of systemic inflammation, an important pathophysiologic contributor to IgAN.

IgAN is thought to arise due to autoimmunity, involving the aberrant activation of both the alternative and mannose-binding lectin (MBL) pathways. Complement activation at both glomerular and systemic levels plays a key role in the pathogenesis and the clinical presentations of IgAN [5–8]. Serum complement levels are surrogate markers of the degree of complement activation, and complement 3 (C3) and complement 4 (C4) are the most widely measured parameters during clinical practice. However, it remains unclear whether serum complement levels can predict outcomes in patients with IgAN. The main objective of this study was to investigate the relationship between serum C3 and C4 levels and the prognosis of patients with IgAN.

## Methods

### Subjects

Participants of this study were enrolled based on the following criteria: biopsy-proven primary IgAN diagnosed between 2009 and 2013; follow-up until 2015 in the Second Affiliated Hospital of Wenzhou Medical University, China; no history of corticosteroid or immunosuppressant treatment before renal biopsy. The exclusion criteria were as follows: patients demonstrating secondary causes of IgAN, such as systemic lupus erythematosus, Henoch-Schönlein purpura, hepatic disease, or ANCA-associated vasculitides. In addition, those with acute interstitial nephritis on renal biopsy, with severe co-morbid diseases, including chronic infectious diseases, diabetic nephropathy, hypertensive nephropathy, or malignancy, were also excluded.

### Collection of clinical variables

Demographic data (age and gender) and clinical features, including systolic and diastolic blood pressure (BP), body weight, body height, as well as serum creatinine, and 24-h urine protein levels were collected at the time of renal biopsy and during follow-up. Mean arterial pressure (MAP) was calculated as diastolic pressure plus one-third of the pulse pressure. Hypertension was defined according to a BP above 140/90 mmHg. Use of medications including immunosuppressant and antihypertensive medications (angiotensin-converting enzyme inhibitor [ACEI] and angiotensin receptor blocker [ARB]) were also recorded. Results from renal biopsy results were graded according to the Oxford Classification of IgAN [9]. Serum creatinine was measured using an enzymatic assay (AU5831 Biochemistry Auto analyzer, Tokyo, Japan) at the time of biopsy, and eGFR was calculated using Chronic Kidney Disease Epidemiology Collaboration equation (CKD-EPI) [10]. Serum C3 (reference range 0.9 - 1.8 g/L) and C4 (reference range 0.1 - 0.4 g/L) levels were measured using a turbidimetric method by a commercial kit (Beckman Coulter IMMAGE, USA). 24-h proteinuria was quantified using a benzethonium chloride method by a biochemistry automatic analyzer (HITACHI 7180, Japan).

### Primary outcome

Since the use of the eGFR doubling as the endpoint requires a longer follow-up period, Levey et al. proposed eGFR decline of 30% as an alternative surrogate endpoint in trails of CKD [11], The primary outcome of this study was defined as an eGFR decrease of more than 30% from the baseline level during follow-up.

### Statistical analysis

Numerical variables were presented as mean (SD) or median with interquartile range (IQR). Variance homogeneity was tested by the Bartlett test. A Welch t-test was used for comparing homogeneous data, while a Wilcoxon rank test was used to compare heterogeneous data. A Kruskal-Wallis rank test was used for the comparison of multiple groups. Categorical variables were presented as percentages and compared by a χ^2^ test. Univariate and multivariate Cox proportional-hazards regression analysis was performed using the *cph* mathed of the *rms* package [12], and the default parameters of the program were used during the multivariate analysis. A nomogram was built for the prediction of three- and five-years survival rate. *P*-values were two-tailed and a value less than 0.05 were considered statistically significant. All analyses were performed using R-software (https://www.r-project.org) and graphics were drawn by *ggplot2* [13].

## Results

A total of 456 cases with primary IgAN were retrospectively identified, and 53 cases were excluded due to secondary IgAN (*n* = 4), malignancy (*n* = 1), acute interstitial nephritis (*n* = 2), diabetic and hypertensive nephropathy (*n* = 5), infectious disease (*n* = 13), incomplete data (*n* = 6), and short follow-up time (<6 months) (*n* = 22). Finally, 403 patients were included in this study.

### Baseline clinical, histopathological features, and follow-up results

Patients’ clinical and histopathological features at the time of renal biopsy are shown in Table 1. The mean age was 37.1 (11.8) years; however, there was a small peak at the age of 58. Approximately 32.6% of patients had hypertension at the time of renal biopsy, and 58.3, 26.1, 13.6, 1.2 and 0.7% patients had stage 1, 2, 3, 4, and 5 chronic kidney diseases (CKD), respectively. The follow-up characteristics are shown in Table 2. The mean duration of follow-up was 33.7 (16.6) months after renal biopsy, and 9.8% (*n* = 39) developed the primary outcome, in which 16 patients developed end stage renal disease. 202 (50.1%) received corticosteroid treatment alone or in combination with other immunosuppressant (GS group), while others received no immunosuppressant treatment (non-GS group). Among this cohort, proteinuria and serum creatinine levels were significant higher in the GS group than those in the non-GS group (for proteinuria, 1.56 g/day [0.94-3.03] versus 0.9 g/day [0.5-1.2], *p* < 0.001; for serum creatinine, 83 μmol/L [59.5-107.2] versus 75 μmol/L [57-95], *p* = 0.02). Most patients received ACEIs or ARBs for blood pressure control and for the reduction of proteinuria.

**Table 1:** Clinical characteristics and the Oxford IgAN classification at the time of renal biopsy in 403 patients with IgAN

Clinical characteristics		Oxford classification (n [%])	
Female (n [%])	223 (55.3)	Mesangial hypercellularity	
Age (years, mean [SD])	37.1 (11.8)	M0	280 (69.4)
Systolic blood pressure (mmHg, mean [SD])	129.7 (18.1)	M1	121 (30.0)
Diastolic blood pressure (mmHg, mean [SD])	81.4 (12.1)	Endocapillary hypercellularity	
MAP (mmHg, mean [SD])	97.6 (13.4)	E0	274 (68)
Hemoglobin (g/L, mean [SD])	125.9 (1 9.0)	E1	127 (31.5)
Serum creatinine (μmol/L, mean [SD])	87.7 (46.3)	Segmental glomerulosclerosis	
eGFR (ml/min/1.73 m2, mean [SD])	94.5 (30.9)	S0	101 (25.1)
24 h urinary protein (g/L, median [IQR])	1.2 (1.5)	S1	300 (74.4)
Serum C3 (g/L, mean [SD])	0.95 (0.19)	Tubular atrophy/interstitial fibrosis	
Serum C4 (g/L, mean [SD])	0.22 (0.07)	T0	294 (73.0)
		T1	84 (20.8)
		T2	23 (5.7)

*SD* standard deviation, *IQR* interquartile range

**Table 2:** Clinical characteristics of participants at the end of follow-up

Clinical characteristics	
Duration of follow-up (months, median [IQR])	35 (11)
Serum creatinine (μmol/L, median [IQR])	78 (41)
UPCR (g/g, median [IQR])	0.5 (1.0)
eGFR (ml/min/1.73m^2^, mean [SD])	91.1 (34.7)
ESRD (n [%])	16 (4.0)
Endpoint event (n [%])	39 (9.8)
Immunosuppressive therapy (n [%])	202 (50.1)
ACEI/ARB (n [%])	255 (87.3)

*SD* standard deviation, *IQR* interquartile range

### The association between serum complement levels and eGFR during follow-up

**Fig. 1:**
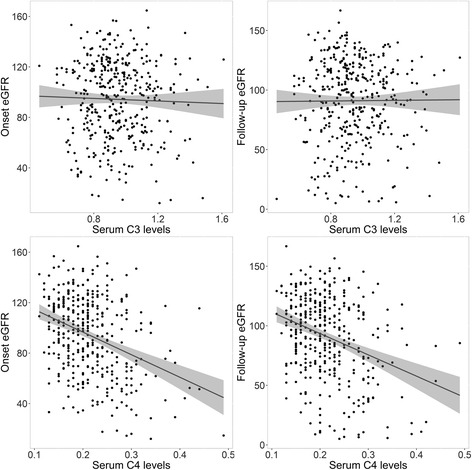
Result from the linear regression analysis of the relationship between serum complement levels and eGFR values. Linear fitting showed that there was no significant correlation between serum C3 levels and eGFR values. Analyses also demonstrated that higher serum C4 levels were associated lower values of eGFR. Grey area outside the fitted line was the confidence interval of the fitted value

We next performed linear regression to investigate whether serum complement levels at the time of renal biopsy were associated with renal function at baseline or at the end of follow-up. There was no significant association between serum C3 levels and eGFR values at renal biopsy (regression coefficient, −4.95, *p* = 0.57) or between serum C3 levels and eGFR at the end of follow-up (regression coefficient, −1.36, *p* = 0.89). However, we observed a significant association between serum C4 levels and eGFR values at renal biopsy (regression coefficient, −171.5, *p* < 0.001) and between serum C4 levels and eGFR values at the end of follow-up (regression coefficient, −163.2, *p* < 0.001) (Fig. 1).

### The association between serum complement levels and histopathological features

**Fig. 2:**
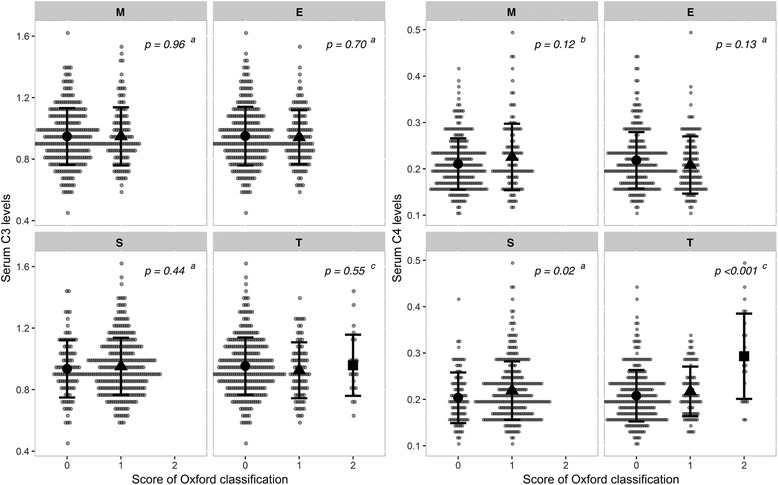
The dot plot showed the relationship between the serum complement levels and the grade of each component of the Oxford classification. Variance homogeneity test was tested by a Bartlett test. ^a^: Welch two sample t-test; ^b^: Wilcoxon rank test; ^c^: Kruskal-Wallis rank test. The mean and standard deviation were showed in the pictures. Abbreviations: E, Endocapillary hypercellularity; M, Mesangial hypercellularity; S, Segmental glomerulosclerosis, T, Tubular atrophy/interstitial fibrosis

We used the Oxford Classification to categorize the pathological findings from all participants, and compared serum complement levels between different groups to evaluate the relationship between complement levels and histopathological features (Fig. 2). We found no association between serum C3 levels and scores using the Oxford classification, however serum C4 levels were significantly associated with scoring of tubular lesions (coefficient 0.27, *p* < 0.001) and segmental glomerulosclerosis (coefficient 0.12, *p* = 0.02).

### Risk factors for the prediction of poor renal prognosis

**Fig. 3:**
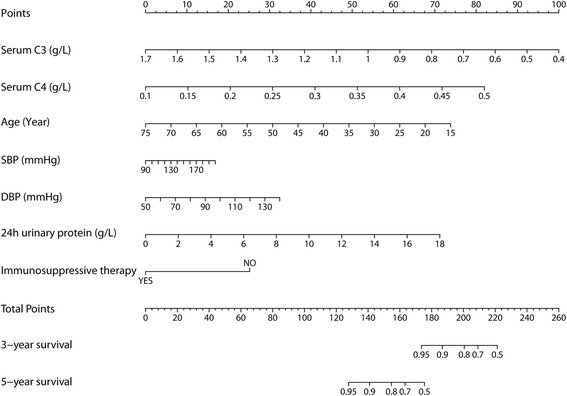
A nomogram to predict the probabilities of three- and five-year event-free survival. Points are assigned for parameters, such as serum C3 and C4 levels by drawing a line upward from the corresponding values to the “Points” line. The sum of these two points, plotted on the “Total points” line, corresponds to the prediction of three- and five-years endpoint events

We performed univariate and multivariate Cox proportional-hazards regression analyses (Table 3). In the univariate analysis, serum C3 (hazard ratio 0.6, 95% confidence interval 0.4–0.9, *p* = 0.03), C4 (hazard ratio 1.5, 95% confidence interval 1- 2.2, *p* = 0.03), as well as age, systolic and diastolic blood pressure, 24-h urine protein, and immunosuppressive therapy were associated with a worse renal prognosis among patients with IgAN. Including those variables with *P* values less than 0.05 in the univariate analysis, multivariate analyses demonstrated that serum C3, C4, as well as age, 24-h urine protein, and immunosuppressive therapy were independent risk factors for renal outcome. For visualization of the role of these risk factors in predicting poor renal outcomes, all variables in the multivariate models were used to construct a nomogram for estimating three- and five- year survival (Fig. 3).

**Table 3:** Univariate and Multivariate Cox proportional-hazard regression analyses for the risk factors of poor outcome

Factors	Univariate Analysis		Multivariate Analysis	
	HR (95%CI)	*p*-value	HR (95%CI)	*p*-value
Age (Year)	0.5 (0.3-0.8)	0.003	0.3 (0.2-0.6)	<0.001
Gender		0.32		-
Female	1		-	
Male	1.4 (0.7-2.6)		-	
Systolic blood pressure (mmHg)	1.5 (1-2.2)	0.04	1.2 (0.6-2.3)	0.52
Diastolic blood pressure (mmHg)	1.5 (1-2.2)	0.05	1.4 (0.7-2.6)	0.32
24 h urinary protein (g/L)	1.4 (1.2-1.6)	<0.001	1.4 (1.1-1.7)	0.003
Serum C3 (g/L)	0.6 (0.4-0.9)	0.03	0.4 (0.2-0.6)	<0.001
Serum C4 (g/L)	1.5 (1-2.2)	0.03	2.4 (1.6-3.8)	<0.001
Immunosuppressive therapy		0.04		<0.001
GS group	1		1	
non-GS group	2.0 (1.1-4.0)		4.0 (1.8-8.8)	

*HR* hazard ratio, *CI* confidence interval

## Discussion

Although risk factors for poor prognosis in patients with IgAN have been reported previously [1, 2], it is still difficult to accurately estimate renal prognosis in these patients. Although serum levels of C3 and C4 frequently fluctuate within the normal range in IgAN patients, studies have increasingly focused on the role of complement in the pathogenesis of IgAN [14–17]. However, the relationship between serum complement levels and the renal prognosis of patients with IgAN remains controversial.

There were studies discussing the relationship between serum C3 levels and the prognosis of IgAN. Kim et al. reported that low serum C3 at the time of renal biopsy was an independent risk factor for poor prognosis [18]. Lbels et al. also found that higher serum C3 at the end of follow up were associated with adverse outcomes, but lower baseline serum C3 levels were unrelated to prognosis [19]. Conversely, Komatsu et al. did not identify any difference in serum C3 levels between patients with severe and mild histopathological lesions, and further suggested that the serum IgA to C3 ratio, rather than C3 levels alone, might be a more appropriate marker for renal outcomes in patients with IgAN [20]. Similarly, another study comparing complement levels between IgAN patients and healthy volunteers did not reveal differences in serum C3 levels [21]. Few have specifically focused on the relationship between C4 and the prognosis of patients with IgAN. Lbels et al. found an independent association between serum C4 levels and patient outcomes, suggesting that these parameters reflect the severity of chronic inflammation [19]. Furthermore, Zhu et al. found that higher C4 levels at the time of renal biopsy were associated with a greater degree of renal injury, including lower eGFR, higher MAP and 24-h urinary protein, and more severe pathological features, and lower C4 levels were related to poor prognosis, a counter-intuitive finding [14]. These studies are all limited in a variety of ways as the authors considered either C3 or C4 alone but not in combination, and this approach may therefore be inadequate for discerning the association between complement levels and renal outcomes.

Our data showed that serum C3 levels were not associated with eGFR at the time of renal biopsy, nor with those at the end of the follow-up, which was consistent with others. On the contrary, we found that serum C4 levels were significantly associated with the S and T scores of the Oxford IgAN classification, and also with eGFR values. Recently, the IgAN Classification Working Group published a new classification of IgAN including crescents, called MEST-C [22]. Our data also showed that serum C4, but not C3 levels, were significantly associated with C score (*P* = 0.04 and *P* = 0.5, respectively).

Complement components are acute phase reactive proteins, and chronic inflammation has been shown to enhance the production of complement components by hepatocytes [23]. Evidence suggests that IgAN is associated with an increase in the activation of the MBL pathway, measured as C4d and MBL deposition in glomerulus. These findings were associated with more severe of symptoms and histopathological performance [24, 25]. Espinosa et al. found that in MBL-activated IgAN, patients had a significantly higher proportion of S1 and T1/T2 scores, but not E1 or M1 scores [24], which was consistent with our observations (Fig. 2). Furthermore, the increase in serum levels of MBL is associated with higher levels of serum complement C4 [26]. These data suggest that elevated levels of C4 may increase kidney damage through the MBL pathway.

Age, systolic blood pressure, diastolic blood pressure, 24 h urine protein are all considered to be classic risk factors of poor renal prognosis. After adjusting for these factors, the risk of adverse prognosis significantly increased by 2.5 times for every one unit decrease in serum C3 levels, and by 2.4 times for every one unit increase in serum C4 levels. Review of the literature suggests that the secretion, activation, and breakdown of serum complements increase during intra-renal inflammation [27]. Furthermore, serum complement levels depend upon their rate of synthesis and breakdown, which differs according to genetic background, age, the severity of disease, and the pathway of activation [21]. It is therefore reasonable that we consider the outcome-predictive effect of serum C3 and C4 together, so that the results may be more reflective of the entire picture of complement activation at the time of assay.

The limitations of our study include its retrospective nature, the short period of follow-up, and the low event number of the primary outcome, all of which potentially lead to bias. Also, there may be significant differences in the doses and the duration of immunosuppressants, the content of which were not included in the analysis. This issue can affect the predictability of serum complement levels for renal prognosis among patients with IgAN. A rigorously designed prospective study is needed to confirm our results.

## Conclusion

In conclusion, increased serum C4 and decreased C3 levels at renal biopsy were associated with poor renal prognosis in patients with IgAN. Analyzing serum C3 and C4 levels in one model can improve the prediction of renal prognosis in these patients.

## Abbreviations

ACEI: Angiotensin-converting enzyme inhibitor
ARB: Angiotensin receptor blocker
BP: Diastolic blood pressure
C3: Serum complement 3
C4: Serum complement 4
CKD: Chronic kidney diseases
CKD-EPI: Chronic Kidney Disease Epidemiology Collaboration equation
E: Endocapillary hypercellularity
eGFR: Estimated glomerular filtration rate
ESRD: End-stage renal disease
HR: Hazard ratio
IgAN: Immunoglobulin A nephropathy
IQR: Interquartile range
M: Mesangium hypercellularity
MAP: Mean arterial pressure
MBL: Mannose-binding lectin
S: Segmental glomerulosclerosis
SD: Standard deviation
T: Tubular atrophy/interstitial fibrosis
UPCR: Urine protein to creatinine ratio
